# Shear Stress Promotes Arterial Endothelium-Oriented Differentiation of Mouse-Induced Pluripotent Stem Cells

**DOI:** 10.1155/2019/1847098

**Published:** 2019-11-15

**Authors:** Yan Huang, Xiaofang Chen, Jifei Che, Qi Zhan, Jing Ji, Yubo Fan

**Affiliations:** ^1^Key Laboratory for Biomechanics and Mechanobiology of Ministry of Education, School of Biological Science and Medical Engineering, Beihang University, Beijing 100083, China; ^2^Beijing Advanced Innovation Center for Biomedical Engineering, Beihang University, Beijing 100083, China; ^3^Beijing Key Laboratory of Rehabilitation Technical Aids for Old-Age Disability, National Research Center for Rehabilitation Technical Aids, Beijing 100176, China

## Abstract

Establishment of a functional vascular network, which is required in tissue repair and regeneration, needs large-scale production of specific arterial or venous endothelial cells (ECs) from stem cells. Previous *in vitro* studies by us and others revealed that shear stress induces EC differentiation of bone marrow-derived mesenchymal stem cells and embryonic stem cells. In this study, we focused on the impact of different magnitudes of shear stress on the differentiation of mouse-induced pluripotent stem cells (iPSCs) towards arterial or venous ECs. When iPSCs were exposed to shear stress (5, 10, and 15 dyne/cm^2^) with 50 ng/mL vascular endothelial growth factor and 10 ng/mL fibroblast growth factor, the expression levels of the general EC markers and the arterial markers increased, and the stress amplitude of 10 dyne/cm^2^ could be regarded as a proper promoter, whereas the venous and lymphatic markers had little or no expression. Further, shear stress caused cells to align parallel to the direction of the flow, induced cells forming functional tubes, and increased the secretion of nitric oxide. In addition, Notch1 was significantly upregulated, and the Notch ligand Delta-like 4 was activated in response to shear stress, while inhibition of Notch signaling by DAPT remarkably abolished the shear stress-induced arterial epithelium differentiation. Taken together, our results indicate that exposure to appropriate shear stress facilitated the differentiation of mouse iPSCs towards arterial ECs via Notch signaling pathways, which have potential applications for both disease modeling and regenerative medicine.

## 1. Introduction

Cardiovascular disease, which is often triggered by endothelial dysfunction, continues to be the leading cause of mortality worldwide. Cell-based therapies have great promise for providing new solutions for treating vascular disease. In particular, induced pluripotent stem cells (iPSCs), cells that are reprogrammed from somatic cells to an embryonic stem cell- (ESC-) like pluripotent state, have been identified as potential candidates for the mass generation of lineage- and patient-specific endothelial cells (ECs) without any ethical concerns.

Differentiation of iPSCs towards ECs *in vitro* can be induced by a variety of methods [1, 2]. The first approach to differentiate ECs from hiPSCs is coculture with stromal cells, usually murine bone marrow-derived stromal cell lines like OP9 or M10B2 [3, 4]. Later on, endothelial lineage-committed cells could also be derived from the formation of 3D cell spheroids in suspension culture referred to as embryoid bodies (EBs) [5, 6]. Furthermore, feeder-free monolayer differentiation methods have been applied to induce ECs from iPSCs. A number of growth factors like vascular endothelial growth factor (VEGF) and basic fibroblast growth factor (bFGF) are typically required to initiate endothelium-oriented differentiation of stem cells [7, 8]. In addition, endothelial progenitor and endothelial differentiation could be efficiently produced from iPSCs via GSK3 inhibition in the absence of exogenous growth factor stimulation [9]. Although all of these approaches have demonstrated differentiation of iPSCs to endothelial lineage, before we can routinely use induced pluripotent stem cell-derived endothelial cells (iPSC-ECs) in human vascular therapies, it is essential to be able to control endothelium-oriented differentiation of iPSCs with high efficiency and reproducibility.

Biophysical cues have recently emerged as key regulators of cell phenotype and tissue morphogenesis. Within the vasculature, shear stress is the dominant physical force experienced by ECs lining the lumen of the vessels. ECs are in direct contact with blood and is therefore exposed to shear stress, which plays critical roles in the development of new blood vessels in both embryos and adults. In embryos, shear stress may be involved in morphogenesis during embryonic development, as blood islands derived from the Flk-1+hemangioblast arise coincidently with the onset of vascular flow [10, 11]. In adults, shear stress is the key to the maintenance of the phenotype, orientation, metabolic activities, and homeostasis of the vascular endothelium [12, 13]. In many *in vitro* studies including ours, it has been demonstrated that the modulation of various shear stress parameters (i.e., magnitude, duration, and pulsatility) can dynamically manipulate EC function and phenotype [11, 14]. Immature and nonlineage-committed endothelial cells, derived from iPSCs, may possess greater inherent plasticity as compared to primary ECs. Physiological levels of shear stress were able to efficiently mature hiPSC-ECs into arterial-like cells in 24 h, thus demonstrating the importance of biomechanical flow on EC subtype specification [15].

Given the crucial role of shear stress in the function of ECs, it is believed that applying shear stress in cell culture could be effective to differentiate stem cells towards the endothelial phenotype. The effect of shear stress on different types of stem cells has been studied previously. Endothelial progenitor cells (EPCs) are derived from the bone marrow and circulate in small numbers in the arterial bloodstream. Suzuki et al. [16] found that 15 dyne/cm^2^ of shear stress augmented the expression of mRNAs encoding CD31 and von Willebrand factor (vWF) as well as the vWF protein. ESCs are capable of differentiating into all mesoderm-derived cell lineages, including endothelial, hematopoietic, and cardiac cell types. Wolfe et al. reported that laminar shear stress magnitudes ranging from 1.5 to 15 dyne/cm^2^ promoted endothelial and hematopoietic differentiation of ESCs [16]. Mesenchymal stem cells (MSCs), also known as mesenchymal stromal cells, were first identified in the bone marrow stroma but are also suggested to be present in other tissues. Many studies have demonstrated that MSCs are able to differentiate into ECs when they are stimulated with shear stress [18–20]. For example, Wang et al. [19] treated MSCs with 15 dyne/cm^2^ of shear stress using a parallel-plate flow system. They found that the cells aligned in the direction of flow; upregulated EC markers including CD31, VE-cadherin, and vWF; enhanced acetylated LDL uptake; and increased tubule formation on Matrigel. The findings of Yuan et al. [20] suggest that high-level shear stress (20 dyne/cm^2^) can induce VEGF production and EC differentiation from MSCs. Our group's previous study also showed that the combined stimulus of VEGF and steady laminar flow stimulation promoted MSC differentiation into ECs [18]. Despite the tremendous progress in differentiation of stem cells exposed to shear stress towards endothelial lineage, the differentiation into arterial and venous endothelial subtypes remains elusive.

It is desirable to induce stem cells towards more specific arterial or venous endothelial phenotype for various research and clinical applications. To promote arterial or venous differentiation, VEGF and PKA activator 8-bromoadenosine-3′:5′-cyclic monophosphate sodium salt (8bromo-cAMP) was introduced to increase the expression of special markers [21, 22]. For example, Flk1-expressing mesoderm cells induced from iPSCs differentiated to venous ECs by adding VEGF alone and to arterial ECs by adding VEGF and 8bromo-cAMP [21]. The endothelial progenitors induced from ESCs differentiated to venous ECs in the absence of VEGF and to arterial phenotype under low concentrations of VEGF [22]. Besides, using fully defined culture conditions developed by combining single-cell RNA sequencing of embryonic mouse endothelial cells with an EFNB2-tdTomato/EPHB4-EGFP dual-reporter human embryonic stem cell line could differentiate human pluripotent stem cells into arterial ECs [23]. But very limited data are available on how these stem cells could be coaxed into arterial or venous ECs under different mechanical factors. ECs exist in different mechanical microenvironments *in vivo*. When the heart beats, arteries mainly deliver well-oxygenated blood from the heart to the various tissues of the body, whereas veins collect and return deoxygenated blood back to the heart. Blood flow results in shear stress experienced by ECs, the magnitude of which is dependent on many *in vivo* factors. In arteries, shear stresses are within the range of 6-40 dyne/cm^2^, whereas in veins, shear stresses are within the range of 0-6 dyne/cm^2^ [24, 25].

To elucidate the effects of shear stress on the vascular differentiation of stem cells and generate large homogenous populations of ECs, in this study, we focused on the impact of different magnitudes of shear stress on the differentiation of mouse iPSCs towards arterial or venous ECs.

## 2. Materials and Methods

### 2.1. iPSC Culture

Induced pluripotent stem cells were induced from Oct4-EGFP mouse (Jackson Laboratory) embryonic fibroblasts using Yamanaka factors [26] as previously described [27]. Single colonies with a high level of GFP expression were picked and maintained on a feeder layer with a passage time of 2 to 3 days. The culture medium for iPSCs was knockout DMEM supplemented with 10% fetal bovine serum (FBS), 10% knockout serum replacement (KSR), 1% GlutaMAX™, 1% NEAA, 1% *β*-mercaptoethanol (all from Thermo Fisher) and 1000 U mL^−1^ LIF (Merck Millipore). All iPSCs expressed Oct4, Sox2, Rex1, and Nanog. These cells maintain the developmental potential to differentiate into advanced derivatives of all three primary germ layers. All the cells were cultured in an incubator at 37°C with saturated humidity and 5% CO_2_.

### 2.2. Application of Shear Stress

Before shear stress treatment, iPSCs were differentiated on fibronectin- (FN-) coated glass slides to promote cell attachment for 3 days in the differentiation medium, consisting of DMEM supplemented with 10% FBS, 10% KSR, 1%GlutaMAX™, 1% NEAA, and 1% *β*-mercaptoethanol.

A parallel-plate flow chamber was used to shear cultured cells as previously described [18, 28, 29]. In brief, cells were seeded on the glass microscope slides. The slides were placed in the flow block such that *τ* = 6*Qμ*/(*wh*^2^), where the shear stress (*τ*) depended on the flow rate (*Q*), viscosity of the media (*μ*), as well as the width (*w*) and height (*s*) of the flow channel. The system was kept at 37°C and equilibrated with 95% humidified air containing 5% CO_2_. Using this system, we applied steady laminar shear stress at 5, 10, or 15 dyne/cm^2^ for 4 h. Then, the cells were cultured under static conditions for 5 d. iPSCs that are continuously cultured under static conditions were considered the static control. To find the optimal concentration of growth factors to induce the endothelium-oriented differentiation of iPSCs, different concentrations of growth factors (25 ng/mL VEGF and 5 ng/mL FGF, 50 ng/mL VEGF and 10 ng/mL FGF, 100 ng/mL VEGF and 20 ng/mL FGF; PeproTech, Rocky Hill, NJ) were added into the medium. To inhibit Notch signaling, a *γ*-secretase inhibitor, 50 *μ*mol/L N-[N-(3,5-difluorophenacetyl)-L-alanyl]-S-phenylglycine *t*-butyl ester (DAPT, Sigma, St. Louis, MO) was used.

### 2.3. MTT Measuring

5 mg/ml MTT was added to cells and mixed by shaking briefly on an orbital shaker. Samples were incubated for 4 h at 37°C after that process. Then, the supernatant was removed, and 150 *μ*L dimethyl sulfoxide was added following 10 min of oscillation. The optical density (OD) of samples was measured at 490 nm using a Thermo Scientific Varioskan Flash multiplate reader (Thermo Inc., Waltham, MA).

### 2.4. CCK-8 Assay

Cells were incubated with CCK-8 solution (Dojindo, Japan) for 3 h at 37°C. Then, the OD value was measured at an absorbance wavelength of 450 nm using a Thermo Scientific Varioskan Flash multiplate reader.

### 2.5. Cytoskeletal Staining

Cells were fixed in 4% paraformaldehyde, then permeabilized with 0.1% Triton X-100 in PBS and blocked in 1% bovine serum albumin. Cells were incubated in Texas red isothiocyanate-conjugated phalloidin (Molecular Probes, Eugene, OR) for 30 min to stain all the F-actin filaments and with DAPI (Sigma) for 5 min to label the nuclei at room temperature as previously described [29]. The fluorescent images were taken under a Leica TCS NT confocal microscope (Wetzlar, Germany).

### 2.6. Reverse Transcription Polymerase Chain Reaction (RT-PCR) Analysis

The mRNA levels of FLK-1, VE-cadherin, EphrinB2, Neuropilin-1, EphB4, COUP-TFII, Prox-1, Notch1, the ligand Delta-like 4 (Dll4), and glyceraldehyde-3-phosphate dehydrogenase (GAPDH) were analyzed using RT-PCR analysis. Total RNA was extracted from cultured cells using TRIzol reagent (Invitrogen, Carlsbad, CA) according to the manufacturer's protocol and quantified using a GeneQuant pro RNA/DNA calculator (Bio-Rad, Hercules, CA). cDNA was synthesized by two-step RT using the reverse transcriptase M-MLV (Takara, Kyoto, Japan), followed by PCR using Taq DNA Polymerase (Fermentas, Ontario, Canada). The forward and reverse sequences of the primers in PCR are listed in Table 1 and synthesized by Sangon Biotech (Shanghai, China). Real-time PCR was performed in an iCycler iQ real-time PCR detection system (Bio-Rad). A 1 *μ*L cDNA sample was added to 5 nmol of each primer, 10 *μ*L of 2× SYBR Green Supermix (Takara) and PCR-grade water to a volume of 20 *μ*L. PCR cycling conditions were as follows: initial 95°C for 30 seconds, then 40 cycles using 95°C for 10 seconds, and 58°C for 35 seconds. Melt curve analysis was performed on the iCycler over the range 55°C to 95°C by monitoring iQ SYBR green fluorescence with increasing temperature (0.5°C increment changes at 10-second intervals). RT-PCR was performed for 30 cycles of 94°C for 30 s, 55°C for 1.5 min, and 68°C for 1 min, with an additional 7 min incubation at 72°C after completion of the final cycle. After PCR amplification, a 10 *μ*L sample of each PCR product was size fractionated by 1.5% agarose gel, stained with ethidium bromide, photographed, and then, scanned using an image acquisition and analysis system (3111, Tanon Inc., Shanghai, China). The GAPDH gene was used as a housekeeping gene. The relative expression of each gene was normalized to GAPDH.

### 2.7. Antibodies and Immunocytochemical Analysis

Cells were fixed in 4% paraformaldehyde, then permeabilized with 0.1% Triton X-100 in PBS, and blocked in 1% bovine serum albumin. Cells were incubated in primary monoclonal antibodies to CD31 (AbDSerotec, Oxford, United Kingdom), vWF (AbDSerotec), or VE-cadherin (Abcam, Cambridge, MA) at a dilution of 1 : 100 for 120 min at room temperature, followed by a tetramethylrhodamine- (TRITC-) conjugated antibody (Zhongshan Goldenbridge Biotechnology, Beijing, China) at a dilution of 1 : 100 for 90 min at room temperature, and finally stained with DAPI for 5 min to label the nuclei at room temperature. The fluorescent images were taken under a Leica TCS NT confocal microscope.

### 2.8. Western Blotting

The protein expression of VE-cadherin, EphrinB2, and Neuropilin-1 was evaluated by western blotting as previously described [29]. Total protein was quantified with a BCA protein assay kit (Dingguo Changsheng, China). Whole-cell protein extracts (20 *μ*g/lane) were separated by SDS-PAGE using a Bio-Rad Mini-PROTEAN Tetra electrophoresis system and transferred to a polyvinylidene difluoride Immobilon-P membrane using a Bio-Rad Mini Trans-Blot System (Bio-Rad Inc., Hercules, CA). Membranes were blocked with bovine serum albumin for 30 min at room temperature, followed by overnight incubation at 4°C with primary antibodies to VE-cadherin, EphrinB2 (Abcam), Neuropilin-1 (Boster Biotechnology, Wuhan, China), or GAPDH (Goodhere Biotechnology, Hangzhou, China) at a dilution of 1 : 500. Primary antibody binding was detected using a HRP-conjugated secondary antibody and super ECL, photographed, and then scanned using the image acquisition and analysis system.

### 2.9. Nitric Oxide Assay

Medium samples were collected from each of the samples and frozen at -80°C. NO content was assayed according the instruction for NO detection kit (Nanjing Jiancheng, China). NO^3-^ was deoxidized to NO^2-^ which then became color compound through Griess diazotization reaction. The absorbance was measured at 550 nm with a spectrophotometer.

### 2.10. Tube-Like Formation

To assess the tube-like formation capacity of cells *in vitro*, a thin layer of Matrigel (BD Bioscience, San Jose, CA) was used to coat the wells of a 96-well plate, followed by seeding with cells directly in a minimal volume of medium. After 24 hours, wells were fixed in 4% paraformaldehyde, and networks were imaged using an Olympus IX71 inverted microscope (Tokyo, Japan).

### 2.11. Statistical Analysis

Each experiment was conducted at least three times. All data were collected from cultures obtained from independent isolations. Statistical analysis was performed using one-way analysis of variance (ANOVA) unless otherwise specified. Tukey's test was used to determine the difference between two groups within the multiple groups. All data were expressed as mean ± SD. Differences were considered significant when *P* < 0.05. All calculations were performed using SPSS (SPSS Inc., Chicago, IL).

## 3. Results

### 3.1. Shear Stress Alone Alters F-Actin Cytoskeleton of iPSCs, but Cannot Affect the Proliferation or Induce the Endothelial Differentiation of iPSCs

**Figure 1:**
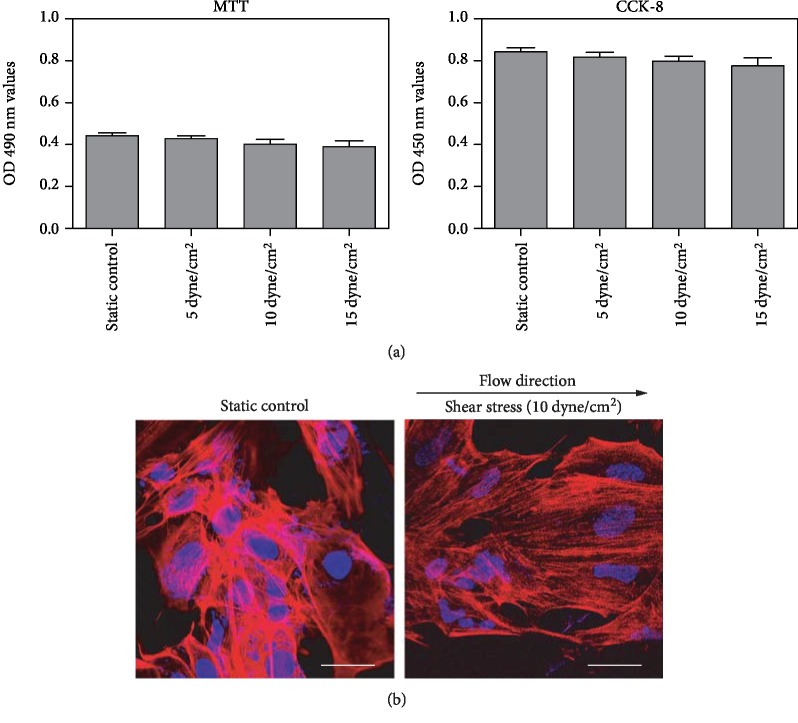
Effect of shear stress on the proliferation and F-actin cytoskeleton of iPSCs. (a) Effect of shear stress on the proliferation of iPSCs measured by MTT and CCK-8 assay. The results are presented as the mean ± SD values, *n* = 3. (b) Alignment (F-actin) of iPSCs in response to shear stress. Cells were incubated in Texas red isothiocyanate-conjugated phalloidin to stain all F-actin filaments (red) and with DAPI to label the nuclei (blue). Scale bar, 25 *μ*m.

In samples exposed to shear stress compared to the static controls, there was no statistical differences detected in the cell viability evaluated by either MTT or CCK-8 analysis (*n* = 3, *P* > 0.05; Figure 1(a)), suggesting that shear stress cannot affect cell proliferation during culture of iPSCs. The confocal image showed that F-actin cytoskeleton of iPSCs cultured under static conditions was sparse and had random fiber orientation and iPSCs exposed to shear stress aligned parallel with the flow direction (Figure 1(b)).

**Figure 2:**
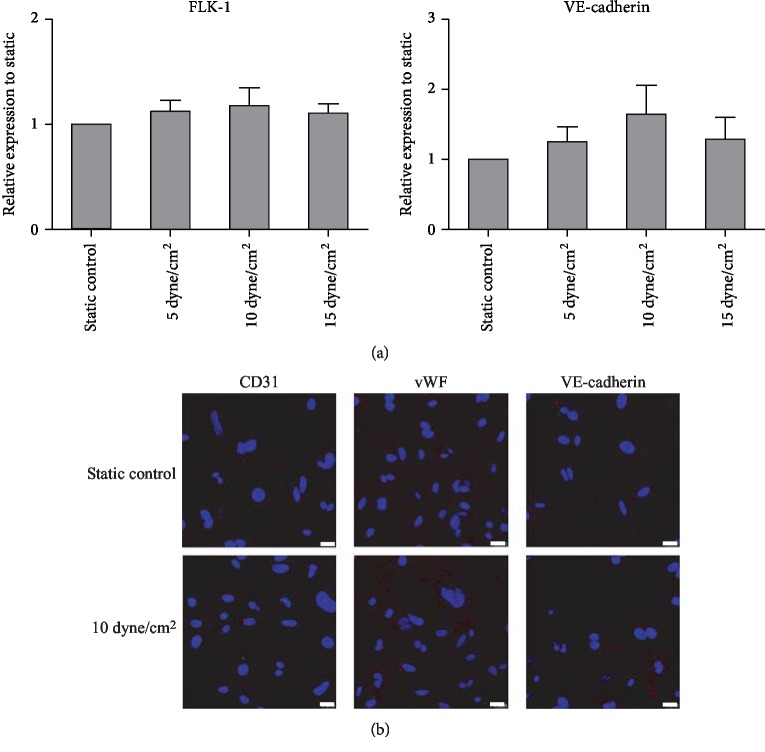
Effect of shear stress alone on the endothelial differentiation of iPSCs. (a) Effect of shear stress application alone on the endothelium-related gene expression evaluated by RT-PCR analysis. The expression of each gene was normalized based on the expression of GAPDH. Results are shown as the mean ± SD values (*n* = 3). (b) Effect of shear stress application alone on the protein expression of CD31, vWF, and VE-cadherin evaluated using immunocytochemical analysis. Red cells in color were taken as positively stained. Scale bar, 25 *μ*m.

To determine the effects of shear stress alone, iPSCs were cultured under static conditions or exposed to shear stress with a different attitude (5, 10, and 15 dyne/cm^2^); then, the mRNA levels of FLK-1 and VE-cadherin were evaluated using RT-PCR. No significant differences were found between the static- and shear-conditioned iPSCs (Figure 2(a)). Immunocytochemical analysis showed that neither static- nor shear-conditioned iPSCs expressed the endothelial marker proteins including CD31, vWF, and VE-cadherin (Figure 2(b)). Thus, shear stress application alone cannot induce the endothelial differentiation of iPSCs.

### 3.2. Endothelial Differentiation of iPSCs Are Promoted by Appropriate Shear Stress in Combination with Growth Factors

**Figure 3:**
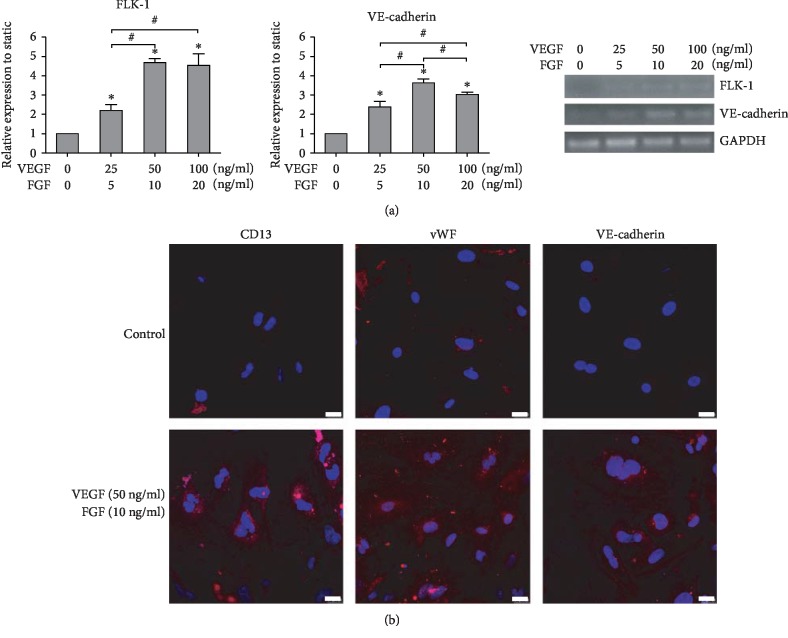
Effect of growth factors on the endothelial differentiation of iPSCs. (a) Effect of growth factors on the endothelium-related gene expression evaluated by RT-PCR analysis. The expression of each gene was normalized based on the expression of GAPDH. Results are shown as the mean ± SD values (*n* = 3). ^∗^*P* < 0.05 compared to the control group; ^#^*P* < 0.05. (b) Effect of growth factors on the endothelial protein expression of CD31, vWF, and VE-cadherin evaluated using immunocytochemical analysis. Red cells in color were taken as positive stained. Scale bar, 25 *μ*m.

To find the optimal concentration of growth factors to induce the endothelium-oriented differentiation of iPSCs, different concentrations of growth factors (25 ng/mL VEGF and 5 ng/mL FGF, 50 ng/mL VEGF and 10 ng/mL FGF, 100 ng/mL VEGF and 20 ng/mL FGF) were added into the medium, and the endothelial cell markers were detected by RT-PCR and immunostaining. The results showed that growth factors could induce the significant endothelial cell marker expression in iPSCs, and the highest values were obtained in the 50 ng/mL VEGF and 10 ng/mL FGF treatment group (*n* = 3, *P* < 0.05, Figures 3(a) and 3(b)).

**Figure 4:**
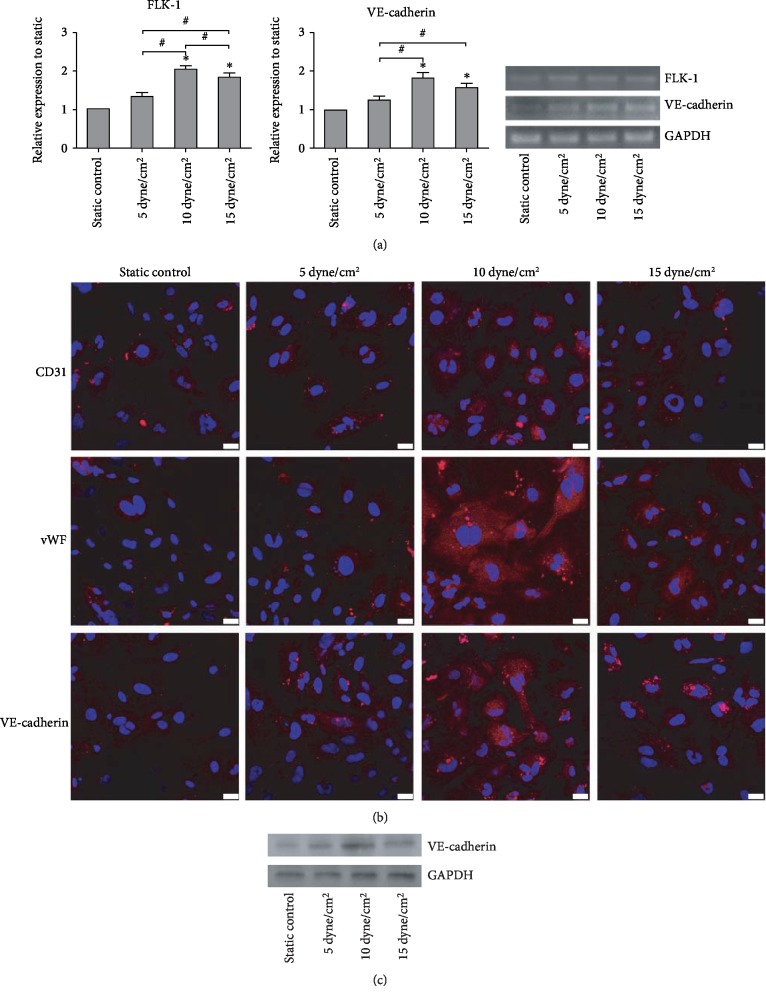
Effect of shear stress on the endothelial differentiation of iPSCs induced by growth factors. (a) Effect of shear stress combined with growth factors on the endothelium-related gene expression evaluated by RT-PCR analysis. The expression of each gene was normalized based on the expression of GAPDH. Results are shown as the mean ± SD values (*n* = 3). ^∗^*P* < 0.05 compared to the static control group; ^#^*P* < 0.05. (b) Effect of shear stress combined with growth factors on the endothelial protein expression of CD31, vWF, and VE-cadherin evaluated using immunocytochemical analysis. Red cells in color were taken as positive stained. Scale bar, 25 *μ*m. (c) Effect of shear stress combined with growth factors on the VE-cadherin protein expression evaluated by western blotting.

To determine if the endothelial differentiation of iPSC scan can be further promoted by the stimulation of shear stress, iPSCs were cultured under shear stress (5, 10, and 15 dyne/cm^2^) combined with 50 ng/mL VEGF and 10 ng/mL FGF. As shown in Figure 4(a), transcriptional expression of the general EC markers including FLK-1 and VE-cadherin increased in a stress-dependent manner for the magnitude of ≤10 dyne/cm^2^. However, mRNA levels started to decrease when the stress magnitude reached 15 dyne/cm^2^ (*n* = 3, *P* < 0.05, Figure 4(a)). To determine if the effects on gene expression led to actual changes towards an endothelial phenotype, the expression levels of CD31, vWF, and VE-cadherin were evaluated using immunocytochemical analysis. CD31, vWF, and VE-cadherin expression levels were significantly higher in shear-conditioned iPSCs as compared to static culture. The highest level of expression appears in the 10 dyne/cm^2^ shear stress treatment group (Figure 4(b)). In addition, the protein expression of VE-cadherin was evaluated by western blotting. VE-cadherin expression levels were strongly increased by shear stress, especially in the 10 dyne/cm^2^ shear stress treatment group (Figure 4(c)).

### 3.3. Shear Stress Increased Arterial Marker Expression

**Figure 5:**
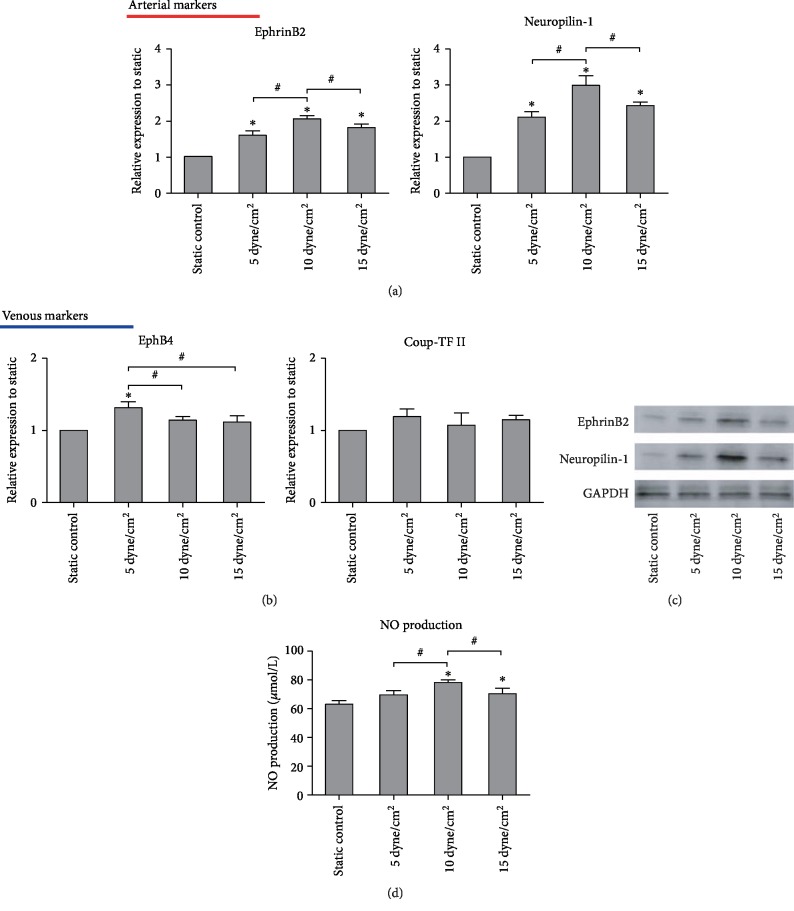
Effect of shear stress on arterial or venous marker expression and NO secretion in iPSCs induced by growth factors. (a) Gene expression of arterial markers EphrinB2 and Neuropilin-1 evaluated by RT-PCR analysis. The expression of each gene was normalized based on the expression of GAPDH. (b) Gene expression of venous markers EphB4 and COUP-TF II evaluated by RT-PCR analysis. The expression of each gene was normalized based on the expression of GAPDH. (c) Protein expression of EphrinB2 and Neuropilin-1 detected by western blotting. (d) Nitric oxide production of shear-conditioned iPSCs. Results are shown as the mean ± SD values (*n* = 3). ^∗^*P* < 0.05 compared to the static control group; ^#^*P* < 0.05.

iPSCs were exposed to shear stress (5, 10, and 15 dyne/cm^2^) with 50 ng/mL VEGF and 10 ng/mL FGF and examined for the gene expression levels of the arterial markers including EphrinB2 and Neuropilin-1. Compared with static culture conditions, the expression of EphrinB2 and Neuropilin-1 in the cells exposed to shear stress increased in a stress-dependent manner for the magnitude of ≤10 dyne/cm^2^. The mRNA levels started to decrease when the shear stress reached 15 dyne/cm^2^ (*n* = 3, *P* < 0.05, Figure 5(a)), whereas the expression of venous markers including EphB4 and COUP-TFII could be detected only in the iPSCs exposed to shear stress of 5 dyne/cm^2^ (*n* = 3, *P* < 0.05, Figure 5(b)). Moreover, the expression of lymphatic marker Prox-1 was not detected in all groups (data not shown). Further, in agreement with the RT-PCR results, the expression of EphrinB2 and Neuropilin-1 detected by western blotting was increased by shear stress, especially in the 10 dyne/cm^2^ shear stress treatment group (Figure 5(c)). Lastly, higher NO production is an important arterial-specific function, so we measured NO secretion in this experiment. Static cultured cells were found to express a low level of NO, but shear stress increased the amount of NO secreted, particularly in the 10 dyne/cm^2^ shear stress treatment group (*n* = 3, *P* < 0.05, Figure 5(d)).

### 3.4. Angiogenesis Network Formation

**Figure 6:**
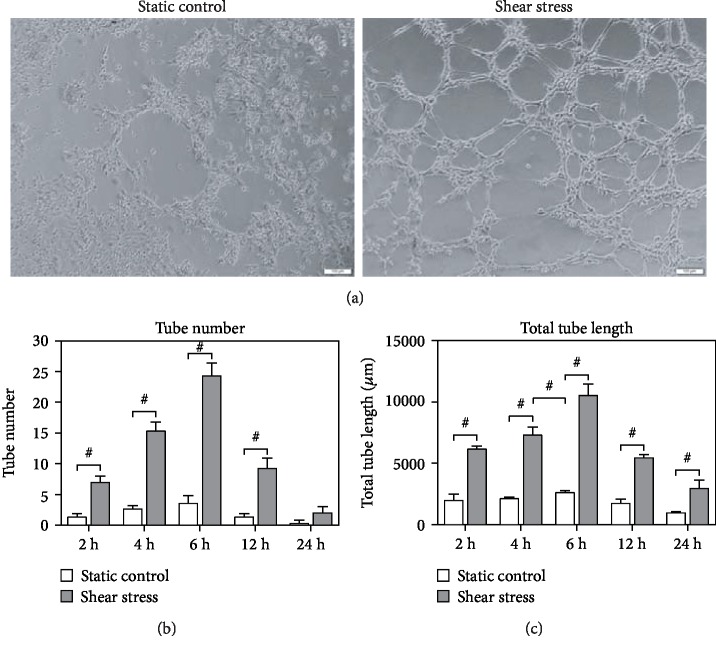
Angiogenesis network formation in shear-conditioned iPSCs. (a) Representative picture of tubular formation on Matrigel for 6 h. Scale bar, 100 *μ*m. (b) The quantification of tube numbers under different conditions. (c) The quantification of tube length under different conditions. Results are shown as the mean ± SD (*n* = 3). The *P* value was calculated using Student's *t*-test. ^#^*P* < 0.05.

Previous studies have shown that arterial lineage ECs can form a more extensive capillary network *in vivo* [30]. Therefore, we examined the angiogenesis network formation. iPSCs were cultured under static conditions, exposed to shear stress of 10 dyne/cm^2^, and then, the cells were cultured on Matrigel for 2 h, 4 h, 6 h, 12 h, and 24 h. We found a statistically significant difference in the total tube numbers between the static- and shear-conditioned iPSCs. iPSCs exposed to shear stress had more tubes compared to the static control group (*n* = 3, *P* < 0.05, Figures 6(a) and 6(b)). We also found that iPSCs exposed to shear stress had a greater tube length compared to those cells under the static culture conditions (*n* = 3, *P* < 0.05, Figures 6(a) and 6(c)). In addition, the maximum tube numbers and the longest tube length could be observed 6 hours after being cultured on Matrigel (Figure 6).

### 3.5. Effect of the Notch Signal Pathway on the Differentiation of Shear-Conditioned iPSCs towards ECs

**Figure 7:**
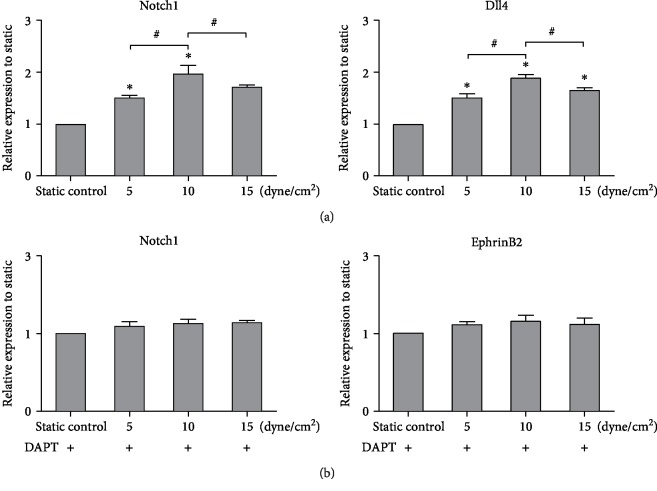
Effect of the Notch signal pathway on the differentiation of shear-conditioned iPSCs towards endothelial cells. (a) Gene expression of Notch1 and Dll4 examined by RT-PCR analysis. (b) Gene expression of Notch1 and EphrinB2 evaluated by RT-PCR analysis. DAPT was used to block Notch signaling. The expression of each gene was normalized based on the expression of GAPDH. Results are shown as the mean ± SD values (*n* = 3). ^∗^*P* < 0.05 compared to the static control group; ^#^*P* < 0.05.

Notch signaling factors are well-known endothelium-oriented regulators [31, 32], so we hypothesized that the Notch signal pathway may play a key role in iPSC differentiation towards ECs. iPSCs were cultured under shear stress (5, 10, and 15 dyne/cm^2^) with 50 ng/mL VEGF and 10 ng/mL FGF. The gene expression levels of Notch1 and Dll4 were examined. As shown in Figure 7(a), shear stress significantly increased the expression of Notch1 and Dll4 as compared with the static culture, especially in the 10 dyne/cm^2^ shear stress group (*n* = 3, *P* < 0.05).

To further confirm the involvement of Notch signal in the differentiation of shear-conditioned iPSCs towards ECs, DAPT was used to block Notch signaling. The addition of DAPT counteracted the effect of shear stress as indicated by the similar levels of Notch1 and EphrinB2 in static and shear stress groups (*n* = 3, *P* < 0.05, Figure 7(b)).

## 4. Discussion

Differentiation of stem cells into the endothelial phenotype would be highly advantageous regarding therapies for peripheral and myocardial ischemia [33]. Establishing iPSC-derived ECs for tissue engineering and therapeutic applications will require methods for promoting not only general endothelium differentiation but also specific EC differentiation (i.e., the arterial or the venous phenotypes) for scale-up applications. The present study showed that shear stress induced an increase in the arterial-oriented differentiation in mouse iPSCs, and stress amplitude of 10 dyne/cm^2^ could be regarded as a proper promoter.

Shear stress is involved in endothelial differentiation during embryonic development and is key to the maintenance of a healthy endothelium. In this study, gene and protein expression analysis demonstrated that shear-conditioned mouse iPSCs have significantly higher levels of the general EC marker FLK-1, VE-cadherin, CD31 and vWF; arterial markers Notch 1 [34], EphrinB2 [35], and Neuropilin-1 [36]; and significantly lower levels or even no expression of venous markers EphB4 and COUP-TFII and lymphatic marker Prox-1 [21] when compared to static controls, indicating an arterial-like, antithrombotic, and anti-inflammatory phenotype [15]. Furthermore, distinct differences were observed in the Matrigel assay, including more tube numbers and longer tube length appearing to be fairly functionally mature cells with an arterial-like phenotype [22]. Additionally, cells could align parallel to the direction of flow, demonstrating flow-sensing ability similar to *in vivo* vessels [37]. Lastly, shear stress upregulated the amount of NO secretion. Since NO production has been implicated in vascular remodeling and angiogenesis [38], our results indicate that the cells were behaving in a physiologically relevant manner. All of these changes in response to shear stress indicated that the application of shear stress promoted iPSC differentiation towards an arterial endothelium phenotype.

The present study showed that a stress amplitude of 10 dyne/cm^2^ was most effective to promote the arterial-oriented differentiation of mouse iPSCs. The expression levels of the general and arterial EC markers were dramatically reduced for shear stress treatments of 15 dyne/cm^2^, which means a higher magnitude shear stress impeded endothelial differentiation. Moghadam et al. [39] hypothesized the existence of a “Goldilocks zone” consisting of an intermediate shear stress (10 dyne/cm^2^) for endothelial differentiation. Consistent with this theory, some studies including our group's previous and present research have shown an increase of endothelial cell marker expression in MSCs at shear stress levels of 10 dyne/cm^2^ [18, 40, 41], while some studies reported that high-level shear stress (15 dyne/cm^2^ or 20 dyne/cm^2^) could increase EC differentiation from MSCs [19, 20]. Besides, effect of shear stress on the arterial EC marker ephrinB2 expression has been reported in ES cell-derived VEGFR2^+^ cells and mature ECs. The results showed that the expression of eprhinB2 in ES cell-derived VEGFR2^+^ cells exposed to shear stress (5, 10, 15, and 20 dyne/cm^2^) increased dose dependently [42] and the expression of eprhinB2 significantly decreased in human umbilical vein ECs and human coronary artery ECs exposed to shear stress (15, 30, and 50 dyne/cm^2^) [43]. Although the exact reason for the discrepancy is unclear, it seems to be the result of differences in the shear apparatus used among studies and be attributable to the difference among mature ECs, immature ECs and stem cells from different sources. The results of our study support the concept that shear stress play a critical role in artery-vein specification during blood vessel development in the embryo.

The present study demonstrated that shear stress causes activation of Notch signaling in mouse iPSCs. Expression of Notch receptor Notch 1 was significantly upregulated in response to shear stress as well as activated the Notch ligand Dll4. More importantly, inhibition of Notch signaling by DAPT remarkably abolished the arterial endothelium-oriented differentiation of shear-conditioned iPSCs. Then, the increased Notch signaling may cause a downstream upregulation of EphrinB2 and Neuropilin-1, while not appreciably affecting venous marker expression [15]. Additionally, COUP-TFII has been found to be expressed specifically in venous ECs, and its function is assumed to maintain venous identity by repressing Notch signaling [42, 44]. Our study indicates that Notch signaling pathways mediates shear-conditioned iPSC differentiation into arterial ECs. Other mechanisms, for example WNT signaling, may be also involved [23]. Clarification of these signaling pathways would provide insights of arterial development and differentiation and will facilitate the understanding of the underlying mechanisms of vascular disease.

We assessed shear stress application alone and the combination of mechanical and biochemical stimuli on the endothelium-oriented differentiation of mouse iPSCs. The results revealed that shear stress application alone cannot induce the endothelial differentiation of iPSC, while the combination of shear stress with growth factors (VEGF and FGF) results in a significant increase of the endothelium-related marker expression compared to biochemical stimuli alone. VEGF is critical for the differentiation of stem cells, including MSCs [45], endothelial progenitor cells [46], embryonic stem cells [47], or iPSCs [48] into ECs *in vitro* [49]. Zhang et al. [23] reported that VEGF and FGF promote arterial endothelial cell differentiation, whereas insulin and bone morphogenetic protein 4 inhibit this process. The failure of shear stress alone to induce EC differentiation may be related to the lack of biochemical factor receptor expression in stem cells [12]. Our results demonstrated the synergistic effect of mechanical and biochemical stimulus in inducing the endothelial function and the expression of endothelial cell markers.

We have developed a physiological pulsatile flow bioreactor which could provide a typical “arterial” level of shear stress *in vitro* [14]. Future work will focus on the analysis correlating the arterial EC differentiation of stem cells with the pulsatile flow conditions. Additionally, although Notch mediates shear-conditioned iPSC differentiation into arterial ECs, the underlying mechanism remains unclear; future research is needed to decipher which downstream signaling pathways are involved.

## 5. Conclusions

Multiple groups have demonstrated the possible therapeutic benefits of using stem cell-derived ECs for treating diseases, including hind limb ischemia, myocardial infarction, and long-term survival after graft implantation [37, 50–52]. This study demonstrated that exposure to appropriate shear stress enhanced mouse iPSCs towards arterial ECs via Notch signaling pathways. The finding could have important implications for the enrichment of ECs towards an arterial-like subtype for therapeutic or research applications in vascular tissue engineering and regenerative medicine.

## Figures and Tables

**Table 1:** Primers for RT-PCR.

Gene	Forward primer	Reverse primer
FLK-1	5′-TGAAATTGAGCTATCTGCCGG-3′	5′-TTTGAAGGTGGAGAGTGCCAG-3′
VE-cadherin	5′-AGATCCCAGAAGAGCTAAGAGGAC-3′	5′-AGAAAAGGAAGAGTGAGTGACCAG-3′
EphrinB2	5′-CTCAACTGTGCCAGACCAGA-3′	5′-CTTGTTGGACCGTGATTCCT-3′
Neuropilin-1	5′-TGTGGGTACACTGAGGGTCA-3′	5′-CAGCAATTCCACCAAGGTTT-3′
EphB4	5′-GGAAACGGCGGATCTGAAATG-3′	5′-TGGACGCTTCATGTCGCAC-3′
COUP-TFII	5′-AGTACTGCCGCCTCAAAAAG-3′	5′-CGTTGGTCAGGGCAAACT-3′
Notch1	5′-GAAACACGCCCAGACCTTGA-3′	5′-ACTCTGCACATGCTGAGGACAC-3′
Dll4	5′-CGAGTGTGTGATTGCCACAG-3′	5′-TCCCATACAGGATGCAATGTAA-3′
GAPDH	5′-TGCACCACCAACTGCTTAG-3′	5′-GATGCAGGGATGATGTTC-3′

## Data Availability

All the data used to support the findings of this study are included within the article. The raw data of RT-PCR, immunofluorescent staining, cell viability, NO assay, and angiogenesis network formation are available from the corresponding author upon request.
